# Supplementary material for: Modeling manual wheelchair propulsion cost during straight and curvilinear trajectories
Source: PLoS One. 2020 Jun 18;15(6):e0234742. doi: 10.1371/journal.pone.0234742 (PMC7302523; doi:10.1371/journal.pone.0234742)
Supplement: S1 Appendix — Descriptions and rationales behind the Straight, Fixed-Wheel Turn, and Alternating Zero-Radius Turns maneuvers. (PDF) [file pone.0234742.s001.pdf]

The importance of this robotic testing platform is the ability to program a repeatable propulsion trajectory. All three canonical maneuvers, as defined in [47], were developed to highlight sources of energy loss from the components and facilitate the limitations of the AMPS. They are described as follows:

1. **Straight Maneuver**

The straight-line trajectory accelerates from rest to 1.0 m/s in 2.5 sec, travels at constant speed for 5.0 sec, then decelerates to a complete stop in 2.5 sec. The total linear distance is 7.5 m.

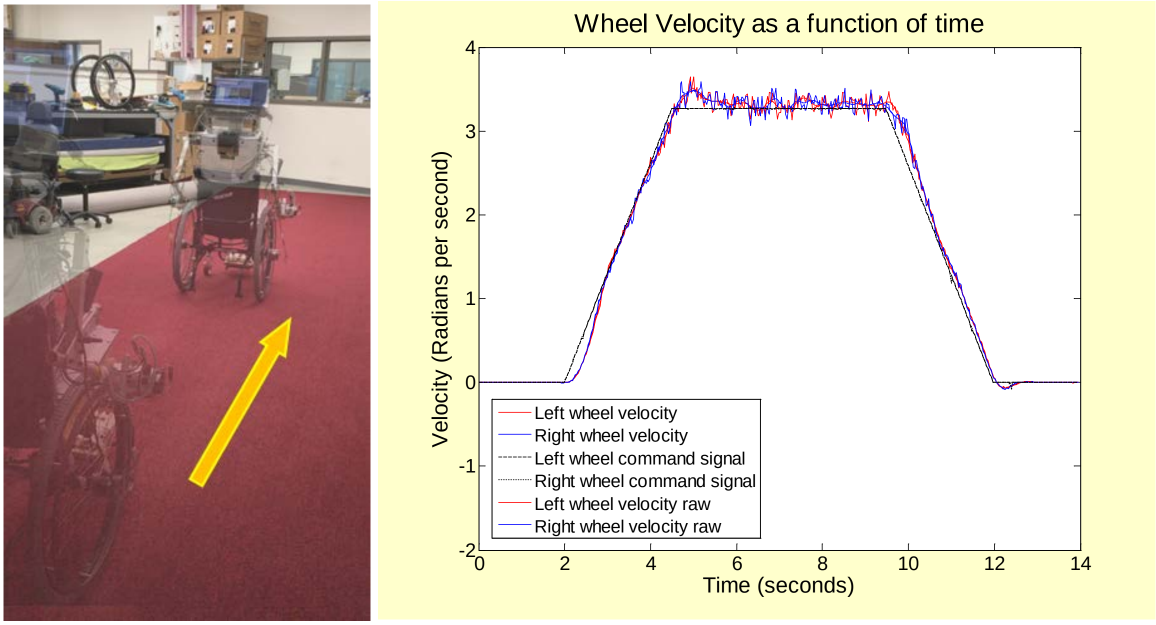

Fig S1. Straight trajectory. (Left) The AMPS traveling across the low-pile carpet during the straight maneuver. (Right) The velocity profile of the straight trajectory. The acceleration phase is from 2.0 sec to 4.5 sec. The steady-state phase is from 4.5 sec to 9.5 sec. The deceleration phase is from 9.5 sec to 12.0 sec.

Rationale: This maneuver is dominated by translational kinetic energy with small contributions from the rotational inertia of the wheels. Therefore, the rectilinear inertia of the system and the rolling resistances of the wheels are highlighted.

2. Fixed-Wheel Turn Maneuver

The fixed-wheel turn starts with one wheel brake engaged on the 'fixed' wheel, and the casters aligned with the direction of travel. The free drive wheel accelerates from rest until the system reaches a yaw rate of 1.56 rad/s for 2.5 sec, holds at steady-state for 5.0 sec, then decelerates to a stop in another 2.5 sec. The rotational displacement is equivalent to a 11.7 rad (or 675°) turn.

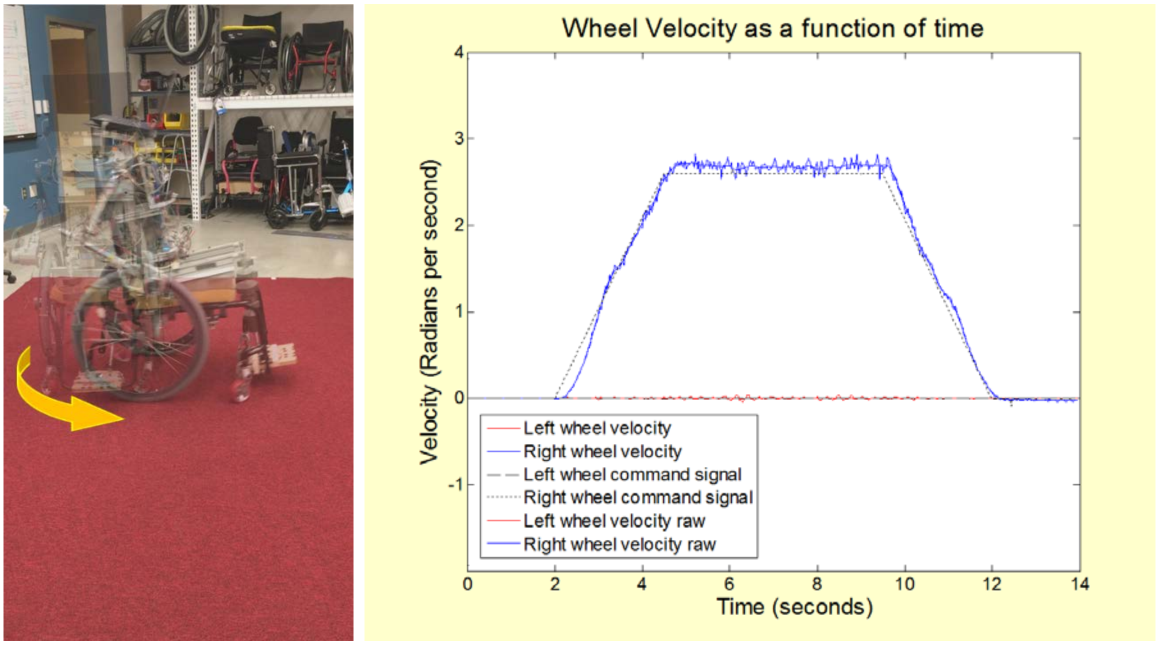

Fig S2. Fixed-wheel turn trajectory. (Left) The AMPS traveling across the low-pile carpet during the fixed-wheel turn maneuver with the left wheel locked. (Right) The velocity profile of the left fixed-wheel turn trajectory. The acceleration phase is from 2.0 sec to 4.5 sec. The steady-state phase is from 4.5 sec to 9.5 sec. The deceleration phase is from 9.5 sec to 12.0 sec. In the left fixed-wheel turn, the left wheel brake is engaged and the left motor is commanded to remain idle.

Rationale: This turning maneuver is designed to highlight the resistive scrub torque of the fixed or stationary drive wheel. Other resistive losses are present with the opposite drive wheel rolling resistance and caster rolling resistance. Aside from initial alignment with the MWC trajectory, the casters do not swivel. Kinetic energy distribution is split between translation of the center of mass and the yaw inertia, with small contributions from the rotational energy of the components.

3. Alternating Zero-Radius Turn Maneuver

The alternating zero-radius turn is a sequential chain of turns about the MWC center of mass. The AMPS starts with casters aligned against the direction of travel. The wheels counter-rotate (one forward, one reverse) until it reaches a yaw rate of 1.56 rad/s in 1.0 sec, then decelerates to a stop in 1.0 sec and rests for a further 1.0 sec. The directions are then reversed and the AMPS rotates back to its original orientation. This process repeats two more times, for a total of six turns. Each turn equates to a 3.14 rad (or 180°) turn, totaling 18.84 rad (1080°) for the entire maneuver. For safety when testing on carpet, the yaw rate is reduced to 1.17 rad/s and held for 1.67 sec in steady-state travel.

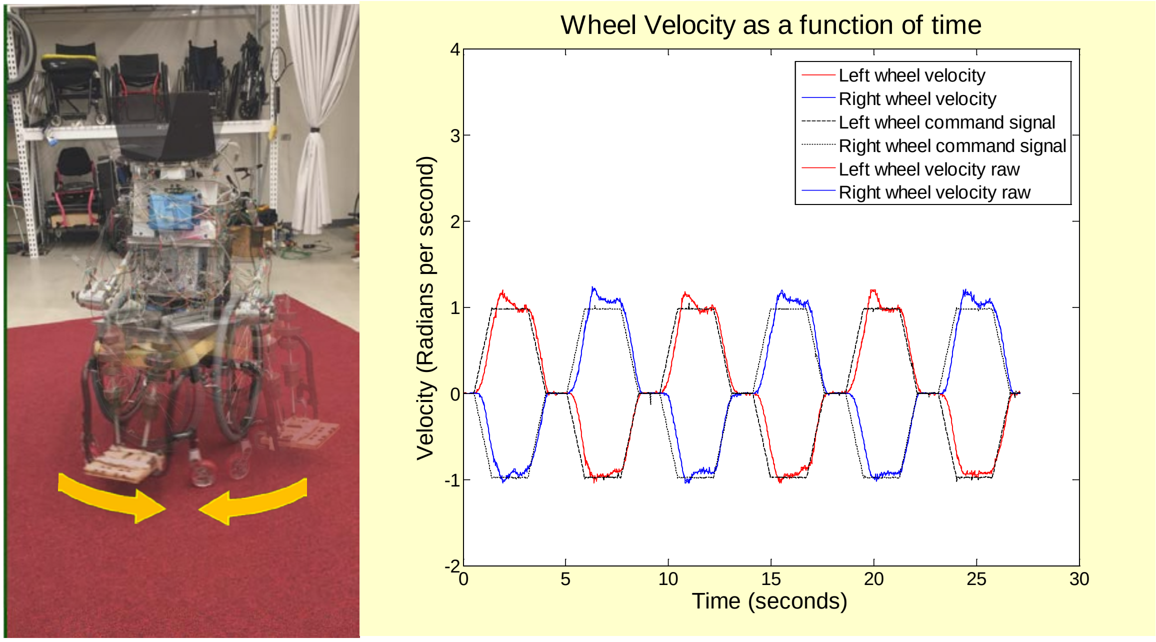

Fig S3. Zero-radius turn trajectory. (Left) The AMPS traveling across the low-pile carpet during the zero-radius turn maneuver. (Right) The velocity profile of the zero-radius turn trajectory. The acceleration phases occur at the start of each trapezoidal velocity command. This maneuver has no true steady-state phase. In this example, the first turn starts with the left wheel traveling forward and the right wheel in reverse, resulting in the chair traveling clockwise. The next turn is counter-clockwise.

Rationale: This maneuver is designed to minimize the translational kinetic energy to highlight the yaw inertia. At the intersection between turns, caster swivel energy loss dominates. Drive wheels experience rolling resistance and resistive scrub torque, though the travel distance is minimized.
