# Supplementary material for: Modeling manual wheelchair propulsion cost during straight and curvilinear trajectories
Source: PLoS One. 2020 Jun 18;15(6):e0234742. doi: 10.1371/journal.pone.0234742 (PMC7302523; doi:10.1371/journal.pone.0234742)
Supplement: Attachment — Submitted filename: PONE-D-20-01476_Reviewer Comments.docx [file pone.0234742.s002.docx]

Manuscript Number: PONE-D-20-01476

Title: Modeling manual wheelchair propulsion cost during straight and curvilinear trajectories

……………………………………………………………………………………………………..

Overall, the paper was well written, and the topic is of interest to the research community who studies manual wheelchairs design, wheelchair biomechanics, Propulsion efficiency.

Abstract:

Line 28: Which predictive variables? Name them.

Introduction:

Lines 41-42: does it mean that modifying the tested wheelchair could leads to lack of sensitivity?

Line 75: Is using non-human operators a limitation for the current study as well? If so, it needs to be discussed in the discussion section.

Line 109: “these”? This is a new paragraph, the topic sentence needs to be rephrased.

Lines 136-143: Using a wheelchair propulsion robot should be mentioned in this section.

The necessity/advantage of using wheelchair propulsion robot needs to be justified in the previous paragraphs.

Overarching approach:

Line 187: “diagram above”? Name the figure.

Methods:

Surface quality should be clearly defined. What type of carpet (high pile/low pile), with/without padding …

Line 203: Cite a reference for the instrumented coast-down cart system.

Equation (1): Why the denominator is 4? Are all 4 wheels considered with the same material and contact surface with the ground?

Equation (2): This is not a new equation it is exactly the same as equation (1).

System-level testing of propulsion cost:

A figure or CAD design of the AMPS could help the readers to better understand the system-level testing used in this study. Figures in the supplemental documents are not clear and informative.

Outcome variable:

Line 288: Propulsion cost over distance, time, or both?

Line 293: How was AMPS power calculated / measured?

Steady state and acceleration phase should be clearly defined.

Line 312: Error!

System loss values:

Line 339: Error!

Results:

Line 395: Yaw inertia

Tables 8-10: Are the how was the mean power calculated? Averaged over different wheel and caster conditions?

Discussion:

Line 466: This is not necessarily correct in all ground conditions!

Figure 3: the diagram does not have a quality. Is it just for one stroke cycle (start-up)?
